# Differential humanistic and economic burden of mild, moderate and severe haemophilia in european adults: a regression analysis of the CHESS II study

**DOI:** 10.1186/s13023-022-02300-1

**Published:** 2022-04-04

**Authors:** Idaira Rodriguez-Santana, Pronabesh DasMahapatra, Tom Burke, Zalmai Hakimi, José Bartelt-Hofer, Jameel Nazir, Jamie O’Hara

**Affiliations:** 1 HCD Economics, The Innovation Centre, Keckwick Lane, Daresbury, UK; 2 Sanofi Genzyme, Cambridge, MA USA; 3 University of Chester, Chester, UK; 4 Sobi, Stockholm, Sweden; 5 Sanofi, Chilly-Marzin, France

**Keywords:** Haemophilia A, Haemophilia B, Direct medical costs, Societal costs, Productivity, Quality of life

## Abstract

**Background:**

The lifelong nature of haemophilia makes patient-centred and societal assessments of its impact important to clinical and policy decisions. Quantifying the humanistic and economic burden by severity is key to assessing the impact on healthcare systems. We analysed the annual direct medical (excluding factor replacement therapy costs) and non-medical costs as well as societal costs and health-related quality of life (HRQoL) of mild, moderate and severe disease among adults with haemophilia A or B without inhibitors in Europe. Participants in the CHESS II study reported their HRQoL, non-medical costs, and work impairment; physicians provided costs and consultation history from the medical chart. Descriptive statistics summarized patient characteristics, costs, and HRQoL scores. Regression models estimated differences in outcomes for moderate and severe versus mild disease, adjusting for age, body mass index, country, comorbidities, weight-adjusted factor consumption and education.

**Results:**

The analytic sample included 707 patients with a mean age of 38 years; the majority of patients had haemophilia A (81%), and 47% had severe disease, followed by moderate (37%) and mild disease (16%). Patients with severe or moderate disease had on average higher direct costs, €3105 and €2469 respectively, versus mild disease. Societal costs were higher for patients with severe and moderate disease by €11,115 and €2825, respectively (all *P* < 0.01). HRQoL scores were also significantly worse for severe and moderate patients versus those with mild disease.

**Conclusion:**

Severity of haemophilia is predictive of increasing economic and humanistic burden. The burden of moderate disease, as measured by direct costs and HRQoL, did not appear to be substantially different than that observed among patients with severe haemophilia.

**Supplementary information:**

The online version contains supplementary material available at 10.1186/s13023-022-02300-1.

## Background

Congenital haemophilia is a rare recessive genetic bleeding disorder characterised by a deficiency or absence of clotting factor VIII (haemophilia A) or factor IX (haemophilia B) affecting primarily males. The severity of haemophilia is determined by the patient’s level of clotting factor activity, where those with < 1 IU/dL are considered to have severe disease, 1 to 5 IU/dL are considered moderate, and > 5 to < 40 IU/dL are considered mild [1]. Haemophilia A is much more common than haemophilia B, with a worldwide prevalence recently estimated in a meta-analysis of national registries to be 17.1 cases per 100,000 males, compared with 3.8 per 100,000 males for haemophilia B [2]. The prevalence of severe disease is approximately 6 cases per 100,000 males for haemophilia A, and 1.1 per 100,000 males for haemophilia B [2].

The clinical burden of haemophilia is persistent, lifelong, and detrimental. Breakthrough bleeding into joints causes disability and pain. Accumulated bleeds over time cause joint deterioration often requiring joint-replacement surgery, particularly in patients with severe disease [3, 4]. The extent of joint damage has been associated with increased health resource utilisation in patients with severe haemophilia, where non-drug-related direct costs are significantly higher for patients with target joints [5]. A joint becomes a target joint when there are three or more spontaneous bleeds within a consecutive 6-month period [1]. Consequently, patients with severe haemophilia suffer long-term sequelae that impact their work productivity, daily functioning, and overall health-related quality of life (HRQoL) [5, 6]. Clotting factor replacement therapy is an inherent part of life for patients with haemophilia in addition to the clinical burden of symptoms. Adherence to treatment is influenced by levels of pain, depression and anxiety [4, 5].

The lifelong nature of haemophilia makes both patient-centred and population-based assessments of disease burden important for clinical and health policy decisions [7]. While the humanistic and economic burden of severe haemophilia has often been studied [8], quantifiable differences in humanistic and economic outcomes among severity levels has not been well characterized in the literature. A recent systematic review highlighted the need for better characterization of the burden of mild haemophilia on HRQoL and costs, primarily citing inconsistent definitions and small samples sizes related to a lack of focus on this population [9]. In order to quantify the differential direct and societal costs and patient-reported HRQoL of mild in relation to moderate or severe disease, we conducted an analysis in adults with haemophilia A or B without inhibitors using the ‘Cost of Haemophilia in Europe: a Socioeconomic Survey’ data set (CHESS II). We excluded the costs of factor replacement therapy from the direct medical cost outcome because treatment costs are known to account for the vast majority of total costs. In order to address a gap in the literature, this study focused specifically on estimating the impact of haemophilia severity on all other medical costs. The costs of factor treatment have been extensively documented in other studies and systematic reviews [10–13].

## Results

### Demographic and clinical characteristics

The CHESS-II data set contained a total of 787 patients managed by 108 haematologists and haematology healthcare providers. Patients with current inhibitors (n = 55) and observations identified as outliers according to *Cook’s Distance* were excluded from the analysis (n = 25) [14]. Data pertaining to direct costs were available for 707 patients (90%), of whom 41% (n = 290) had EQ-5D-5L responses and 40% (n = 286) had available information to calculate societal cost. The smaller patient samples with information on societal costs and HRQoL were comparable in terms of demographic and clinical characteristics to the larger cohort. Demographic and clinical characteristics were generally similar across the analytic samples. Patients with available EQ-5D-5L and societal costs were subsets of the sample with direct costs (Table 1). The mean age of the entire analytic sample was 38 years (age range 18–86), half (56%, n = 399) had normal BMI (body mass index; between 18.5 and 24.9 kg/m^2^), and slightly more than half had no comorbidities (59%, n = 420). The three most common comorbidities were smoking, fatigue and anaemia. Overall, most patients were from Italy (39%, n = 279), Spain (32%, n = 225), or the UK (11%, n = 76).

**Table 1:** Demographic and clinical characteristics of the analytic sample from CHESS II

Characteristic	Direct costs Sample, n = 707	Societal costs Sample, n = 286	HRQoL (EQ-5D-5L) Sample, n = 290
**Age, mean (SD)**	38.4 (14.2)	38.5 (14.7)	38.5 (14.7)
**BMI, n (%)**			
Underweight	12 (2)	0	0
Normal weight	399 (56)	166 (58)	169 (58)
Overweight	276 (39)	113 (40)	114 (39)
Obese	20 (3)	7 (20)	7 (2)
**Country, n (%)**			
Italy	279 (39)	119 (42)	120 (41)
Spain	225 (32)	113 (40)	112 (39)
United Kingdom	76 (11)	14 (5)	16 (6)
France	68 (10)	34 (12)	36 (12)
Germany	49 (7)	6 (2)	6 (2)
Romania	10 (1)	0	0
**Haemophilia type, n (%)**			
A	574 (81)	245 (86)	250 (86)
B	133 (19)	41 (14)	40 (14)
**Severity, n (%)**			
Mild	110 (16)	44 (15)	46 (16)
Moderate	264 (37)	87 (30)	90 (31)
Severe	333 (47)	155 (54)	154 (53)
**Comorbidities, n (%)**			
0	420 (59)	175 (61)	178 (61)
1	157 (22)	66 (23)	65 (22)
≥ 2	130 (18)	45 (16)	47 (16)
Treatment*, n (%)			
*** Overall***	***n* = *707***	***n* = *286***	***n* = *290***
No treatment	261 (37)	91 (32)	94 (32)
On-demand	87 (12)	32 (11)	35 (12)
Prophylaxis	359 (51)	163 (57)	161 (56)
*** Mild***	***n* = *110***	***n* = *44***	***n* = *46***
No treatment	77 (70)	30 (68)	30 (65)
On-demand	7 (6)	0	1 (2)
Prophylaxis	26 (24)	14 (32)	15 (33)
*** Moderate***	***n* = *264***	***n* = *87***	***n* = *90***
No treatment	184 (70)	61 (70)	64 (71)
On-demand	25 (9)	8 (9)	9 (10)
Prophylaxis	55 (21)	18 (21)	17 (19)
*** Severe***	***n* = *333***	***n* = *155***	***n* = *154***
No treatment	0	0	0
On-demand	55 (17)	24 (15)	25 (16)
Prophylaxis	278 (83)	131 (85)	128 (84)
1-year factor replacement therapy consumption, IU/kg, mean (SD)	1232.4 (2211.1)	1635.5 (2646.9)	1583.7 (2624.8)
**Annual bleeding rate, n (%)**			
*** Overall***	***n* = *707***	***n* = *286***	***n* = *290***
0	88 (12)	26 (9)	26 (9)
1 to 5	538 (76)	217 (76)	218 (75)
≥ 5	105 (15)	43 (15)	46 (16)
*** Mild***	***n* = *110***	***n* = *44***	***n* = *46***
0	30 (27)	9 (20)	9 (20)
1 to 5	79 (72)	35 (80)	37 (80)
≥ 5	1 (1)	0	0
*** Moderate***	***n* = *264***	***n* = *87***	***n* = *90***
0	33 (13)	9 (10)	9 (10)
1 to 5	191 (72)	69 (79)	71 (78)
≥ 5	40 (15)	9 (10)	11 (12)
*** Severe***	***n* = *333***	***n* = *155***	***n* = *154***
0	24 (7)	8 (5)	8 (5)
1 to 5	254 (76)	113 (73)	111 (73)
≥ 5	55 (17)	34 (22)	34 (22)
**Number of problem joints, n (%)**			
*** Overall***	***n* = *707***	***n* = *286***	***n* = *290***
0	445 (63)	177 (62)	177 (61)
≥ 1	262 (37)	109 (38)	113 (39)
***Mild***	***n* = *110***	***n* = *44***	***n* = *46***
0	34 (77)	34 (77)	36 (78)
≥ 1	10 (23)	10 (23)	10 (22)
*** Moderate***	***n* = *264***	***n* = *87***	***n* = *90***
0	168 (64)	58 (67)	58 (64)
≥ 1	96 (36)	29 (33)	32 (36)
*** Severe***	***n* = *333***	***n* = *155***	***n* = *154***
0	185 (56)	85 (55)	83 (54)
≥ 1	148 (44)	70 (45)	70 (46)

Percentages may not sum to 100 due to rounding

*BMI* body mass index,; *SD* standard deviation

*“No treatment” category could include patients treated with alternative therapies such us desmopressin or antifibrinolytics

The majority of patients had haemophilia A (81%, n = 574), and approximately half of the patient pool had severe disease (47%, n = 333), followed by those with moderate (37%, n = 264) or mild disease (16%, n = 110). Half of the patient pool were receiving prophylactic factor replacement therapy (51%, n = 359), which was much more frequent among those with severe disease (83%, n = 278). Most patients experienced between 1 and 5 bleeds in the previous 12 months (76%, n = 538; see Table 1). Annual bleeding rate (ABR) and number of problem joints [15] (see "Methods") were consistent with disease severity, as 27% of patients with mild disease had no bleeds in the past year (n = 30) and 77% had no problem joints (n = 34). Among patients with moderate or severe disease, only 13% and 7% (n = 33 and 24, respectively) had no bleeds, and 64% and 56% (n = 168 and 185, respectively) had no problem joints.

**Fig. 1:**
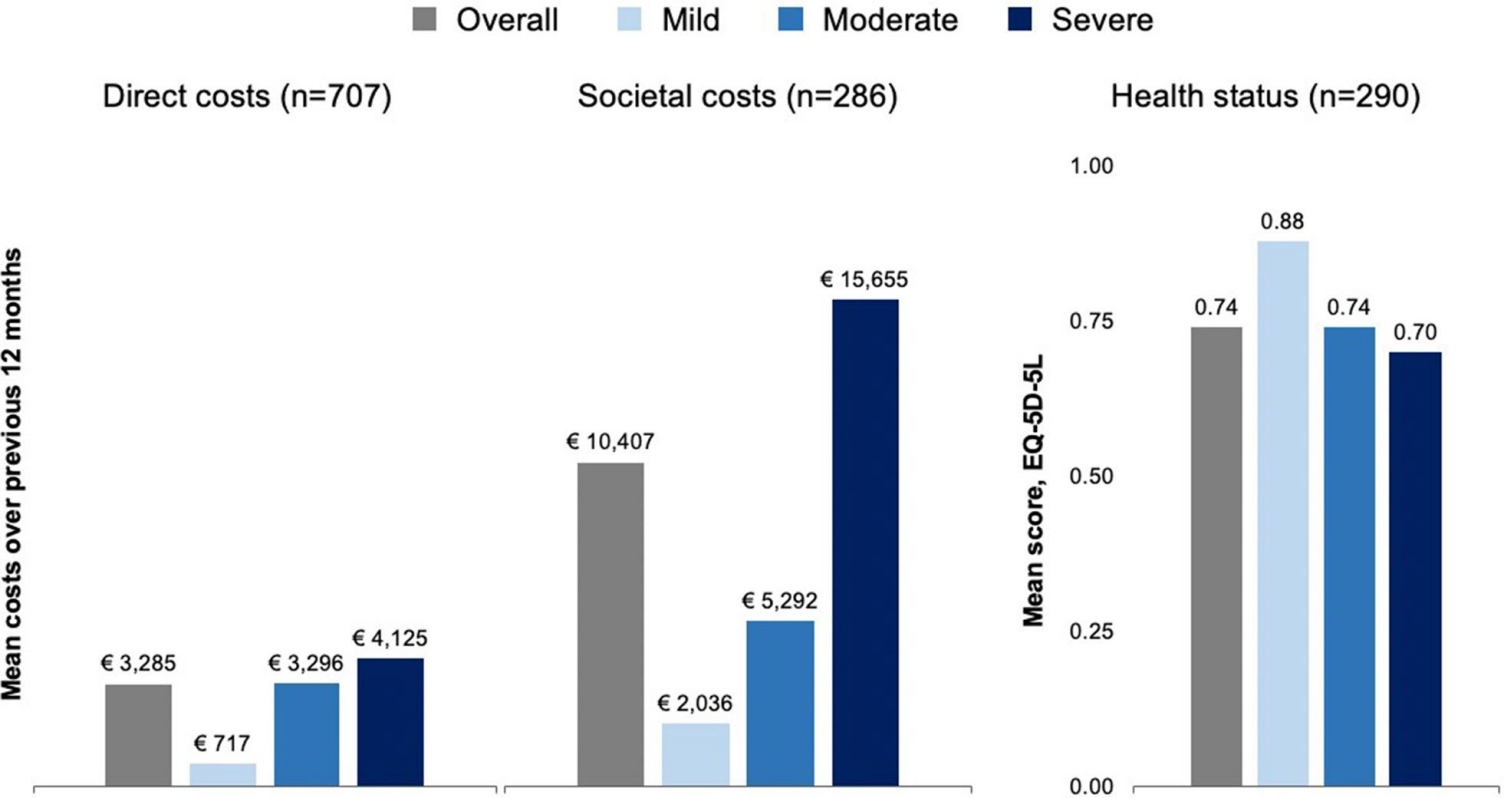
Descriptive direct and societal costs and health status (EQ-5D-5L) overall and by severity. Direct and societal costs are captured at patient level for a period of 12 months

In the descriptive assessment, mean unadjusted annual direct medical cost (excluding factor replacement therapy costs) across all severities was €3285 (SD, €3845). Mean patient-reported HRQoL based on the EQ-5D-5L was 0.74 (SD, 0.22) out of a possible maximum of 1, and mean societal costs, were €10,407 (SD, €15,915). On average, patients with severe haemophilia incurred higher mean annual direct costs (€4125) than those with moderate (€3296) or mild (€717) disease, and a lower mean EQ-5D-5L score (0.70) than those with moderate (0.74) or mild disease (0.88). The societal costs were directionally consistent with those of direct costs, but the difference was markedly greater across levels of severity (Fig. 1).

When analysed by treatment strategy, patients receiving on-demand factor replacement therapy had higher direct and societal costs than those receiving prophylaxis, and the lowest HRQoL scores (Additional file 1: Appendix Table A1). Direct and societal costs were the lowest for patients with mild haemophilia overall, with results for those with mild disease by treatment type potentially biased by a very low sample receiving on-demand treatment. Among patients with severe disease, those receiving on-demand treatment had the highest direct and total societal costs, and the lowest EQ-5D-5L scores. Direct and societal costs were highest in Spain and Italy (countries which also contributed the majority of patients to the sample). As expected, all costs increased with more bleeds and problem joints (Additional file 1: Appendix Table A1).

### Direct and societal haemophilia-related costs by severity

**Fig. 2:**
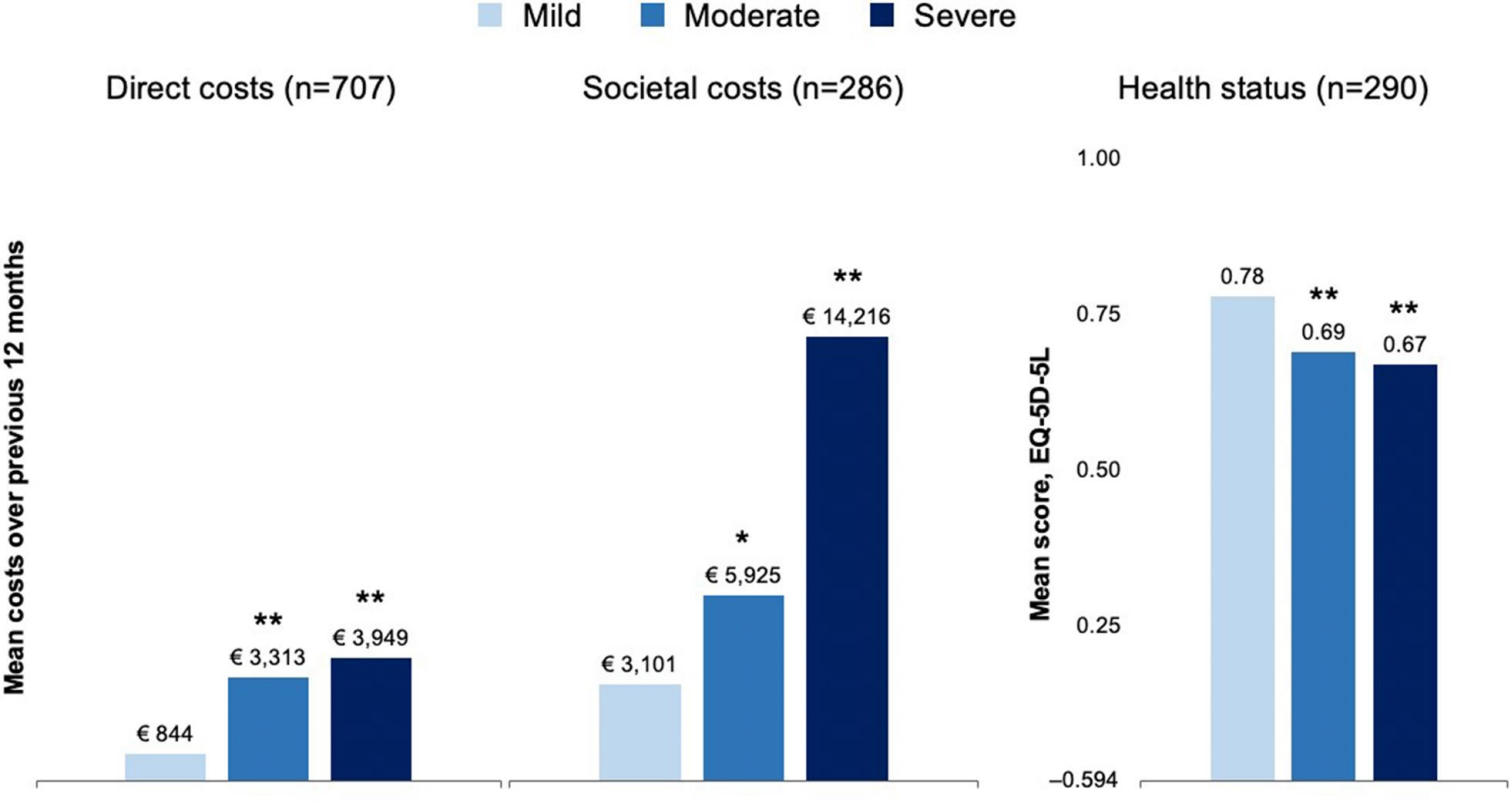
Predicted direct and medical costs and health status (EQ-5D-5L) for mild versus moderate or severe haemophilia. *P < 0.01, **P < 0.001 vs mild disease. Cost models adjusted for haemophilia severity (base outcome: mild), age, BMI, country ( base outcome: Germany), comorbidities (base outcome: 0 comorbidities) and weight-adjusted factor consumption; health status model also adjusted for education level (base outcome: primary). Direct medical and societal costs are captured at patient level for a period of 12 months

The cost regression models controlling for haemophilia severity, age, BMI, country, comorbidities and weight-adjusted clotting factor consumption showed significantly lower direct medical and societal costs for patients with mild versus moderate or severe disease (all *P* < 0.01; Fig. 2). On average, patients with severe or moderate disease would be expected to incur higher annual direct costs of €3105 and €2469, respectively, than those with mild disease. Average societal costs were higher for patients with severe or moderate haemophilia by €11,115 and €2825, respectively, than those for patients with mild disease. The impact of having ≥ 2 comorbidities incurred €1750 greater direct costs as compared to having none, and €5736 greater societal costs (Table 2). The impact of weight-adjusted clotting factor consumption on direct and societal costs was very close to zero and not statistically significant, having been analysed to evaluate the potential impact of access to treatment on other cost elements. Large differences were observed between countries, with significantly higher mean direct and societal costs in Italy, Spain and France compared with Germany. Regression model estimates and goodness of fit tests are provided in Additional file 1: Appendix Table A2.

**Table 2:** Average marginal effects for annual direct medical and societal costs and HRQoL scores (EQ-5D-5L)

Model parameter	Direct costs Sample, n = 707	Societal costs Sample, n = 286	HRQoL (EQ-5D-5L) Sample, n = 290
**Severity, vs Mild**			
Moderate	€ 2469 *P* < *0.001*	€ 2825 *P* < *0.01*	−0.089 *P* < *0.001*
Severe	€ 3105 *P* < *0.001*	€ 11,115 *P* < *0.001*	−0.105 *P* < *0.001*
**Age, years**	€ 14	€ 86	0.001
**Body mass index, kg/m**^**2**^	€ −48.32	€ 881 *P* < *0.01*	−0.003
**Country, vs Germany**			
Italy	€ 1643 *P* < *0.001*	€ 8727 *P* < *0.001*	−0.060
Spain	€ 3274 *P* < *0.001*	€ 12,362 *P* < *0.001*	−0.035
France	€ −633 *P* < *0.05*	€ 5063	0.031
United Kingdom	€ 1478 *P* < *0.001*	€ −319	−0.007
Romania	€ −956 *P* < *0.05*	NA	NA
**Comorbidities, vs none**			
1	€ 547	€ 1616	−0.063 *P* < *0.01*
≥ 2	€ 1750 *P* < *0.001*	€ 5736 *P* < *0.05*	−0.111 *P* < *0.01*
**Factor consumption per kg**	€ 0.198 *P* < *0.001*	€ 1.18 *P* < *0.01*	−5.34 × 10^−6^
**Education, vs Primary**			
Secondary/vocational	–	–	0.04
Tertiary			0.09

Cost models adjusted for haemophilia severity (base outcome: mild), age, BMI, country (base outcome: Germany), comorbidities (base outcome: 0 comorbidities) and weight-adjusted factor consumption; health status model also adjusted for education level (base outcome: primary)

*NA* not available

Direct medical and societal costs are captured at patient level for a period of 12 months

Statistical significance is indicated in italics: *P* < 0.001*; P* < 0.01*; P* < 0.05. *P* > 0.05 for all other differences

### Humanistic burden of haemophilia by severity

The HRQoL regression model controlling for haemophilia severity, age, BMI, country, comorbidities, education level, and weight-adjusted clotting factor consumption showed significantly better mean EQ-5D-5L scores for patients with mild disease compared with moderate or severe disease (both *P* < 0.001; Table 2). Mean predicted HRQoL for patients with mild disease was 0.78 (95% CI: 0.73–0.82; Fig. 2), which was 13% lower for patients with severe disease (−0.105; Table 2) and 11% lower (−0.089) for those with moderate disease. The impact of having either 1 or ≥ 2 comorbidities was significant compared with having none, and as with the cost models, the impact of weight-adjusted clotting factor consumption was approximately zero. Regression model estimates and goodness of fit tests are provided in Additional file 1: Appendix Table A2.

## Discussion

This analysis of the European CHESS II study cohort quantified the economic and humanistic burden of haemophilia across levels of severity in adult patients without inhibitors. Disease severity was a significant predictor of direct medical and societal costs and patient reported HRQoL, with societal costs particularly high in severe patients, suggesting greater indirect costs and productivity losses.

While all outcomes were the least favourable for patients with severe disease, a significant humanistic and economic burden is also shown for patients with moderate disease. Mean societal costs for patients with moderate was double that for patients with mild haemophilia. Only 1% of patients with mild disease had ≥ 5 bleeds in the past year (N = 707), compared to15% and 17% of those with moderate or severe disease. Similarly, at least 1 problem joint was observed in 23% of patients with mild disease, compared to 36% and 44% of patients with moderate and severe haemophilia respectively. While the occurrence of bleeding and problem joints in patients with moderate disease was more similar to those in patients with severe disease, the reported treatment strategy was similar to that observed in patients with mild disease, where 70% of those with mild and 70% of those with moderate disease were reported to have no clotting factor replacement therapy, compared to none of the severe patients (all severe patients having reported on-demand or prophylactic haemophilia treatment). As such, mean adjusted direct medical costs (excluding factor replacement therapy costs) were nearly 3 times higher for moderate versus mild disease (€3313 vs €844), and 3.7 times higher for severe vs mild disease (€3949 vs €844).

HRQoL scores of patients with moderate haemophilia were also more closely approximated those of with severe disease, where mean estimates were 11% and 13% lower than in patients with mild disease respectively. Regression adjusted EQ-5D-5L scores ranged from 0.67 to 0.78 across severities in our study. In contrast the EQ-5D-3L norm country-values ranged from 0.86 to 0.92 for Germany, Italy, France, Spain and the United Kingdom [16]. These informal comparisons were consistent with adult normative index values for the EQ-5D-5L reported from Germany (0.90) and Spain (ranging from 0.74 to 0.98 for adults 80–89 and 18–29, respectively) [17, 18].

Results also show statistically significant differences in costs and HRQoL outcomes between countries. These differences are probably attributable to differences in sample sizes and large structural differences such as health system and disease management strategies, state support for professional caregivers, and other societal factors. Consistent with reports of long-term treatment outcomes [19–21], this study showed that patients with severe disease receiving prophylactic therapy had lower costs and improved HRQoL compared with those receiving on-demand treatment. Patients with moderate or mild disease with no reported clotting factor usage in the study period appeared to have better HRQoL scores and lower costs than patients currently on on-demand or prophylaxis regimens, though this may be attributable to less severe phenotypes within these groups. Of note, the impact of weight-adjusted clotting factor therapy consumption on costs and health status was negligible, nearly zero. This may reflect the greater likelihood of patients with more severe disease receiving more factor replacement therapy, mostly as prophylaxis, demonstrating the impact of prophylactic treatment on reducing the use of health care resources and subsequent costs. Prophylaxis has been associated with fewer hospitalizations, fewer surgical procedures, and less time lost from work than on-demand treatment among adults [19, 21, 22].

Findings of this study should be interpreted in the context of certain strengths and limitations. This work evaluated detailed direct medical and societal costs, and patient-reported HRQoL among a cohort of European male adults with haemophilia A or B without inhibitors from both a patient and societal perspective. Patients with haemophilia A or B were analysed together, to evaluate the burden of haemophilia in general. Nonetheless, the inclusion of haemophilia type as a control variable was not found to be significant. This was a retrospective analysis of an existing observational data set constructed from questionnaires completed by patients and haemophilia healthcare providers. Direct medical costs were based on unit costs assigned to health care services based on medical consultations identified in the patients’ medical records, and may not have captured consultations that were not shared with the provider and not entered in the chart. While patient-reported outcomes are particularly valuable in the context of burdensome, lifelong conditions such as haemophilia, data collection may have been influenced by a selection bias in participation and completion of the questionnaires. Despite the completion of the patient questionnaire being voluntary, the subset of patients with information on societal costs and HRQoL was similar to the larger cohort of participants. To further account for the possible selection bias, we reported descriptive results as recorded by those participating in the CHESS II study and performed rigorous regression analyses to adjust for relevant covariates including haemophilia severity, age, BMI, comorbidities, factor replacement consumption and education level. However, it is possible that unmeasured factors might have had an impact in the estimation of results, which is a common limitation of empirical studies. We also excluded outlier responses that exhibited potentially excessive influence. Recording of clinical outcomes such as bleeding events and joint metrics from patients’ medical charts may be more straightforward than a formal clinical joint assessment requiring imaging studies. The use of the EQ-5D-5L, compared to the EQ-5D-3L, enabled us to quantify a broader of range of HRQoL responses.

## Conclusions

This study illustrates the humanistic and economic burden of increasing levels of disease severity among European adults with haemophilia A and B without inhibitors. We show the substantial burden of moderate disease in the continuum of haemophilia severity. The burden of moderate patients, especially direct costs and HRQoL, is not substantially different to that observed in severe patients. While the majority of studies focus on severe patients, this study addresses a gap in the literature by documenting the burden across levels of severity by means of descriptive and multivariate regression analysis.

Future work may further dissect the burden of patients with moderate disease and the need to mitigate the disparate costs to these patients, their families, and society.

## Methods

We conducted a retrospective analysis of the humanistic and economic burden of adult haemophilia by level of severity (mild, moderate or severe) using the CHESS II dataset, the design and methods of which have been reported previously [8]. Briefly, CHESS II is a cross-sectional study of male adults (≥ 18 years old) with haemophilia A or B, with or without inhibitors, in six European countries (France, Germany, Italy, Spain, the United Kingdom, and Romania); however, this analysis only included data from patients without inhibitors. The study consisted of two questionnaires collecting information from patients and their haemophilia healthcare providers. The treating physician completed a web-based patient record form collecting information about the patient’s medical history and consultations. Patients completed a paper-based questionnaire collecting information on self-reported health status (HRQoL), non-medical costs, and work impairment. Physician-reported record form was available for all participants, while the patient questionnaires were only returned on a voluntary basis (response rate of 42%). CHESS II data was collected between October 2018 and March 2019.

### Outcome measures

Our objective was to understand the differential impact of the level of haemophilia severity on direct medical and societal costs, and patient-reported HRQoL as measured by the 5-level EuroQol-5-dimensions (EQ-5D-5L; www.euroqol.org). Haemophilia-related direct medical costs included physician consultation visits, hospitalisations, surgical procedures, tests and examinations, assistive medical devices (e.g., crutches), over-the-counter self-medication, and costs for professional caregiving (time and hourly cost). Direct non-medical costs included travel expenses for haemophilia-related care, qualifying government support, and alternative therapies. Indirect costs comprised work-productivity impairment based on hours worked per week, absenteeism, informal care costs and early retirement. Societal costs were defined as the sum of all of direct medical, non-direct medical and indirect costs. Direct medical and societal perspective costs were captured at patient level and covered a 12-month period. This analysis focused on the costs of haemophilia-related care excluding factor replacement therapy costs. Haemophilia factor treatment costs have been largely documented in other studies [10–12]. Direct medical and societal costs not associated with haemophilia care were excluded. Costs were calculated for each country, applying country-specific unit costs for each type of haemophilia-related resource use (please see costing detail in Additional file 1: Appendix Table A3).

The EQ-5D-5L instrument comprises five domains: mobility, self-care, usual activities, pain/discomfort and anxiety/depression with 5 levels of severity (“no problems,” “slight problems,” “moderate problems,” “severe problems,” or “extreme problems”). A health state index utility score was derived through an amalgam of the five responses, with scores ranging from 0 (equivalent to “dead”) to 1 (“perfect health”), though scores of less than zero (“worse than dead”) may be derived [23]. UK population norms were used across all sample for comparability purposes.

### Statistical analysis

Descriptive statistics summarised demographic and clinical characteristics, direct medical and societal costs, and EQ-5D-5L. Outcomes were assessed overall and by relevant covariates, including haemophilia severity determined by physician-reported endogenous factor VIII or IX values, i.e. mild (> 5–40%), moderate (1–5%) or severe (< 1%), age, BMI (in kg/m^2^; underweight, < 18.5; normal weight, 18.5 to < 25; overweight, 25 to 30; obese, > 30), country, education (primary, secondary/vocational, tertiary), haemophilia treatment strategy (no treatment, on-demand, or prophylaxis), annualised clotting factor consumption (IU/kg), number of comorbidities (excluding haemophilia-related conditions; 0, 1, ≥ 2), annual bleeding rate (ABR; 0, 1–5, ≥ 5 bleeds in the previous 12 months), and number of problem joints (defined as chronic joint pain and/or limited range of movement due to compromised joint integrity, such as chronic synovitis and/or haemophilic arthropathy; 0 or ≥ 1) [15]. Haemophilia treatment strategy (none, on-demand or prophylaxis) was based on the physician-reported total clotting factor usage (IU) in the previous 12 months. Alternative treatments such as desmopressin were not included. Details of the control variables are provided in Additional file 1: Appendix Table A4.

Regression models estimated cost outcomes for patients with moderate or severe disease with respect to mild using a generalised linear model with Gamma distribution and log-link function. The average marginal effects were computed to ascertain the effect of each covariate on the outcome of interest. For direct and societal costs, four models were tested, controlling for (1) severity only; (2) severity, age, BMI, and country; (3) severity, age, BMI, country, and number of comorbidities; and (4) severity, age, BMI, country, comorbidities, and weight-adjusted factor consumption. Model 4 demonstrated the best goodness of fit (i.e. lowest Akaike information criterion and Bayesian information criterion) and was used in all analyses. Differences in health status scores from the EQ-5D-5L were estimated using a Tobit model bounded between −0.594 and 1.0; average marginal effects were used to show the effect of the covariate on the bounded outcome variable. The same four models described above were tested for HRQoL and in this case, Model 4 also demonstrated the best goodness of fit and was used in all analyses.

Clotting factor consumption was used as control variable and served as a proxy for treatment strategy and to account for patient access to haemophilia factor replacement therapy. A categorical variable for haemophilia treatment strategy and interactions with severity levels were explored but discarded due to multicollinearity issues (e.g., > 80% of severe patients were receiving prophylaxis). In order to estimate the burden of disease across levels of severity in the most generalisable manner, we excluded clinical outcomes from the regression model (although these variables are still explored descriptively), as there is a positive relationship between disease severity and increasing bleeds and problem joints. Patients with current inhibitors or a history of inhibitors in the previous 12 months were excluded. Statistical significance was determined at the 5% alpha level (*P* < 0.05). No imputation of missing values was performed, patients with missing responses were excluded from the analysis. All analyses were performed using STATA® 16 (StataCorp LLC, College Station, Texas; www.stata.com).

## Supplementary Information

**Additional file 1.** **Table A1**: Summary of costs and health status scores by demographic and clinical covariates. **Table A2**: Regression model results, predicted estimates (standard error). **Table A3**: Cost components used in the CHESS II study. **Table A4**: Control variable definitions and data sources

## Data Availability

The data that support the findings of this study may be available from HCD Economics, Ltd but restrictions apply to the availability of these data, which were used under license for the current study, and so are not publicly available. Data may be available from the authors upon reasonable request and with permission of HCD Economics Ltd.

## Abbreviations

ABR: annual bleeding rate
BMI: body mass index
EQ-5D-5L: 5-level EuroQol-5-dimensions
HRQoL: Health-related quality of life
