# Supplementary material for: Differential humanistic and economic burden of mild, moderate and severe haemophilia in european adults: a regression analysis of the CHESS II study
Source: Orphanet J Rare Dis. 2022 Apr 4;17:148. doi: 10.1186/s13023-022-02300-1 (PMC8981861; doi:10.1186/s13023-022-02300-1)
Supplement: Supplementary file 1 — Additional file 1. Table A1: Summary of costs and health status scores by demographic and clinical covariates. Table A2: Regression model results, predicted estimates (standard error). Table A3: Cost components used in the CHESS II study. Table A4: Control variable definitions and data sources [file 13023_2022_2300_MOESM1_ESM.docx]

APPENDIX

Table A1. Summary of costs and health status scores by demographic and clinical covariates

| **Characteristic** | **Direct costs, n=707 (€)** | **Societal costs, n=286 (€)** | **EQ-5D-5L, n=289 (€)** |
| --- | --- | --- | --- |
| **Country, mean (SD)**  Italy  Spain  United Kingdom  France  Germany  Romania | 3,338 (3,479)  4,743 (4,800)  2,458 (2,150)  761 (720)  1,641 (2,780)  505 (786) | 9,566 (14,084)  13,495 (18,197)  817 (758)  8,781 (16,104)  526 (322)  NA | 0.70 (0.24)  0.74 (0.21)  0.78 (0.16)  0.86 (0.14)  0.83 (0.13)  NA |
| **Comorbidities, n (%)**  0  1  ≥2 | 2,502 (3,205)  3,735 (3,885)  5,271 (4,806) | 8,382 (14,515)  11,674 (17,055)  16,426 (17,942) | 0.80 (0.20)  0.69 (0.21)  0.62 (0.24) |
| **Treatment, n (%)**  ***Overall***  No treatment  On-demand  Prophylaxis  ***Mild***  No treatment  On-demand  Prophylaxis  ***Moderate***  No treatment  On-demand  Prophylaxis  ***Severe***  No treatment  On-demand  Prophylaxis | 2,498 (3,485)  4,531 (4,412)  3,556 (3,842)  682 (573)  501 (362)  881 (839)  3,257 (3,893)  3,652 (3,477)  3,262 (3,223)  –  5,443 (4,723)  3,864 (4,025) | 3,772 (6,011)  14,937 (17,700)  13,222 (18,133)  1,812 (1,692)  –  2,514 (2,961)  4,735 (7,068)  6,454 (8,701)  6,660 (8,383)  –  17,765 (19,127)  15,269 (19,420) | 0.81 (0.20)  0.69 (0.19)  0.71 (0.23)  0.92 (0.11)  0.88 (0.88)  0.79 (0.21)  0.76 (0.21)  0.73 (0.17)  0.71 (0.12)  –  0.67 (0.20)  0.71 (0.24) |
| **Annual bleeding rate, n (%)**  0  1 to 5  ≥5 | 1,034 (1,685)  3,433 (4,440)  7,386 (6,921) | 1,784 (3,294)  9,192 (14,516)  21,755 (21,049) | 0.91 (0.14)  0.76 (0.20)  0.56 (0.24) |
| **Number of problem joints, n (%)**  0  ≥1 | 2,136 (2,722)  5,237 (4,617) | 6,987 (13,696)  15,962 (17,674) | 0.81 (0.18)  0.63 (0.23) |

BMI, body mass index; NA, not available; SD, standard deviation.

Direct medical and societal costs are captured at patient level for a period of 12 months.

Cost in Euros, year 2020.

Table A2. Regression model results, predicted estimates (standard error)

| **Model Parameter** | **Direct costs, n=707 (€)** | **Societal costs, n=286 (€)** | **Health status, n=290 (€)** |
| --- | --- | --- | --- |
| **Severity, vs Mild**  Moderate  Severe | 1.37 (13.82) *P<0.001*  1.54 (16.3) *P<0.001* | 0.65 (3.21) *P<0.01*  1.52 (7.51) *P<0.001* | –0.17 (–3.30) *P<0.01*  –0.19 (–3.76) *P<0.001* |
| **Age, years** | 0.004 (1.56) | 0.01 (1.44) | 0.001 (1.21) |
| **Body mass index, kg/m^2^** | –0.01 (–1.24) | 0.08 (2.97) *P<0.01* | –0.005 (–0.79) |
| **Country, vs Germany**  Italy  Spain  France  United Kingdom  Romania | 0.74 (3.53) *P<0.001*  1.16 (5.53) *P<0.001*  –0.55 (–2.44) *P<0.05*  0.69 (3.04) *P<0.01*  –1.02 (–2.28) *P<0.05* | 1.92 (7.28) *P<0.001*  2.22 (8.35) *P<0.001*  1.48 (3.51) *P<0.001*  –0.24 (–0.70)  NA | –0.10 (–1.45)  –0.06 (–0.83)  0.06 (0.76)  –0.01 (–0.16)  NA |
| **Comorbidities, vs None**  1  ≥2 | 0.18 (1.92)  0.49 (4.94) *P<0.001* | 0.16 (0.96)  0.48 (2.32) *P<0.05* | –0.10 (–3.01) *P<0.01*  –0.17 (–3.58) *P<0.001* |
| **Factor consumption per kg** | 5.98 x 10^-5^ (4.19) *P<0.001* | 1.08 x 10^-4^ (3.51) *P<0.001* | –8.80 x 10^-6^ (–1.36) |
| **Education, vs Primary**  Secondary/vocational  Tertiary | – | – | 0.05 (0.80)  0.14 (2.00) *P<0.05* |
| **Constant**  Observations  R-squared  RMSE | 5.90 (16.04) *P<0.001*  707  0.284  3283.53 | 3.36 (4.61) *P<0.001*  286  0.248  14608.81 | 1.04 (5.37) *P<0.001*  290  0.368 (Pseudo R-squared)  0.0576 (sigma, SE) |

Cost models adjusted for haemophilia severity (base outcome: mild), age, BMI, country ( base outcome: Germany), comorbidities (base outcome: 0 comorbidities) and weight-adjusted factor consumption; health status model also adjusted for education level (base outcome: primary).

NA, not available; RMSE, root mean square error; SE, standard error.

Statistical significance indicated in italics: *P<0.001; P<0.01; P<0.05; P>0.05* for all other differences.

Table A3. Cost components used in the CHESS II study

| **Outcome** | **Component category** | **Measured element** |
| --- | --- | --- |
| **Direct medical costs (excluding factor replacement therapy costs)** | Hospitalisations (Reported by the treating physician) | Day case |
|  |  | Outpatient (ie, for planned treatments) |
|  |  | Inpatient, including length of stay |
|  | Surgical procedures (Reported by the treating physician) | Number and type of surgeries |
|  |  | Length of stay |
|  |  | Time spent in intensive care |
|  | Consultant visits (Reported by the treating physician) | Haematologist |
|  |  | Other specialties |
|  | Tests and examinations (Reported by the treating physician) | Blood tests |
|  |  | Other tests and examinations |
|  | Professional caregiver (Reported by patient) | Hourly wage |
|  |  | Hours per week |
| **Direct non-medical costs** | Alternative and Complimentary Therapies (Reported by patient) | Number of visits |
|  |  | Cost per session |
|  | Travel costs (Reported by patient) | Car |
|  |  | Public transport |
|  | Requirement for aids / equipment (Reported by patient) | Walking aids |
|  |  | Home adjustments |
|  | Transfer Payments (Reported by patient) | Entitlement per month |
| **Indirect costs** | Work productivity impact (Reported by patient) | Absenteeism |
|  |  | Early retirement / Stopped working |
|  | Caregiver burden (Reported by patient) | Hours per week |

Note: Country-specific unit cost sources were used to cost direct medical costs, information about cost data sources is available under request. Information on direct non-medical costs and indirect costs were reported by patients in the patient survey (country-average unit costs were used if patient information was not available.). The most recent estimate for each specific unit cost was used, all costs were converted to 2020 Euros.

Table A4. Control variable definitions and data sources

| **Variable** | **Data source (physician/patient)** | **Detail** |
| --- | --- | --- |
| Haemophilia type | Physician | haemophilia A, haemophilia B |
| Severity | Physician | Severe (levels of Factor VIII or IX <1%), Moderate (levels between 1%-5%) and Mild (levels between 5% and 40%) |
| Bleed frequency | Physician | Annual bleed rate (ABR) derived from physician-reported major plus minor bleeds in the previous 12 months:  No Bleeds, 1-5 Bleeds, 5+ Bleeds. |
| Problem joints  (areas of chronic synovitis) | Physician | Problem Joint can be defined as having chronic joint pain &/or limited range of movement due to compromised joint integrity (i.e. chronic synovitis &/or haemophilic arthropathy): None, ≥ 1 |
| Therapy regimen | Physician | Prophylaxis, on-demand, or no treatment based on physician-reported total IU usage in the past 12 months.  No treatment category could include patients treated with alternative therapies such us desmopressin or antifibrinolytics. |
| Clotting factor consumption | Physician | Total IU/kg usage in the past 12 months (weight adjusted) |
| Country | Physician | France, Italy, Germany, Spain, UK and Romania; |
| Age | Physician | Age 18-86 |
| Education | Patient | No education/primary; secondary/vocational; tertiary |
| Body mass index (kg/m^2^) | Physician | Underweight (under 18.5), normal weight (18.5 to 25 kg), overweight (25 to 30 kg), and obese (over 30 kg). |
| Concomitant conditions | Physician | Number of comorbidities the patient is experiencing at index date (haemophilia-related comorbidities are excluded): None, One, Two or more  List of comorbid conditions: myocardial infarction; stroke; atrial fibrillation; venous thrombosis; disseminated intravascular coagulation; pulmonary embolism; arterial embolism; portal vein thrombosis; renal vein embolism; intestinal ischemia; renal vascular disease; acute cerebrovascular disease; angiodysplasia; attention deficit hyperactivity disorder / ADHD; autism spectrum disorder; obsessive compulsive disorder; fatigue; smoking; drug dependence; alcohol dependence; gingivitis; obesity; type I diabetes; type II diabetes mellitus; human immunodeficiency virus; hepatitis B; hepatitis C; anaemia; osteoarthritis; osteoporosis, any other (open field) |
